# Comparative genomics of 11 complete chloroplast genomes of Senecioneae (Asteraceae) species: DNA barcodes and phylogenetics

**DOI:** 10.1186/s40529-019-0265-y

**Published:** 2019-08-22

**Authors:** Andrew Wanyoike Gichira, Sheila Avoga, Zhizhong Li, Guangwan Hu, Qingfeng Wang, Jinming Chen

**Affiliations:** 1 Key Laboratory of Aquatic Botany and Watershed Ecology, Wuhan Botanical Garden, Chinese Academy of Sciences, Wuhan, 430074 China; 2 University of Chinese Academy of Sciences, Beijing, 100049 China; 3 Sino-Africa Joint Research Center, Chinese Academy of Sciences, Wuhan, 430074 China; 4 Key Laboratory of Plant Germplasm Enhancement and Specialty Agriculture, Wuhan Botanical Garden, Chinese Academy of Sciences, Wuhan, 430074 China

**Keywords:** *Senecio*, *Dendrosenecio*, Endemic, Chloroplast genome, Codon usage, Microsatellites, DNA barcodes, Phylogenomics

## Abstract

**Background:**

Majority of the species within Senecioneae are classified in *Senecio*, making it the tribe’s largest genus. Certain intergeneric relationships within the tribe are vaguely defined, with the genus *Senecio* being partly linked to this ambiguity. Infrageneric relationships within *Senecio* remain largely unknown and consequently, the genus has undergone continuous expansion and contraction over the recent past due to addition and removal of taxa. *Dendrosenecio*, an endemic genus in Africa, is one of its segregate genera. To heighten the understanding of species divergence and phylogeny within the tribe, the complete chloroplast genomes of the first five *Senecio* and six *Dendrosenecio* species were sequenced and analyzed in this study.

**Results:**

The entire length of the complete chloroplast genomes was ~ 150 kb and ~ 151 kb in *Dendrosenecio* and *Senecio* respectively. Characterization of the 11 chloroplast genomes revealed a significant degree of similarity particularly in their organization, gene content, repetitive sequence composition and patterns of codon usage. The chloroplast genomes encoded an equal number of unique genes out of which 80 were protein-coding genes, 30 transfer ribonucleic acid, and four ribosomal ribonucleic acid genes. Based on comparative sequence analyses, the level of divergence was lower in *Dendrosenecio*. A total of 331 and 340 microsatellites were detected in *Senecio* and *Dendrosenecio,* respectively. Out of which, 25 and five chloroplast microsatellites (cpSSR) were identified as potentially valuable molecular markers. Also, through whole chloroplast genome comparisons and DNA polymorphism tests, ten divergent hotspots were identified. Potential primers were designed creating genomic tools to further molecular studies within the tribe. Intergeneric relationships within the tribe were firmly resolved using genome-scale dataset in partitioned and unpartitioned schemes. Two main clades, corresponding to two subtribes within the Senecioneae, were formed with the genus *Ligularia* forming a single clade while the other had *Dendrosenecio, Pericallis, Senecio* and *Jacobaea*. A sister relationship was revealed between *Dendrosenecio* and *Pericallis* whereas *Senecio,* and *Jacobaea* were closely placed in a different clade.

**Conclusion:**

Besides emphasizing on the potential of chloroplast genome data in resolving intergeneric relationships within Senecioneae, this study provides genomic resources to facilitate species identification and phylogenetic reconstructions within the respective genera.

**Electronic supplementary material:**

The online version of this article (10.1186/s40529-019-0265-y) contains supplementary material, which is available to authorized users.

## Background

Senecioneae, the largest tribe in the family Asteraceae, has over 160 genera with more than 3000 species, and new genera continue to be added (Chen et al. 2011; Nordenstam et al. 2009). The tribe is prominent for its size, and rich morphological and ecological diversity. It is mostly dominated by annual and perennial herbs, while the rest constitute shrubs, vines, trees, and epiphytes. It has a near cosmopolitan distribution, with southern Africa being one of its key diversity hotspot zones (Pelser et al. 2007). Majority of the species in the tribe are placed in *Senecio* L., making it one of the largest genera of angiosperms, with over 1250 species (Nordenstam et al. 2009). *Senecio* is characteristically diverse in morphology, life-history, growth forms, and thus, it has been markedly linked to the incongruous phylogenetic relationships within the tribe (Pelser et al. 2007). Its members are generally distinguished by style branches truncate with short sweeping hairs, separated stigmatic lines and sometimes with a median hair pencil, and with ecaudate anther bases and balusterform filament collar (Nordenstam 2007; Pelser et al. 2007).

Over the years, the genus has been under constant re-evaluation and reclassification, and until now, comprehensive infrageneric relationships are yet to be established. Consequently, numerous species have in the past been segregated as new genera mostly based on morphological, anatomical, and chromosomal variations (Jeffrey and Chen 1984; Jeffrey et al. 1977). One of such segregate genera is *Dendrosenecio* (Hauman ex Hedb.) B. Nord., upgraded by Nordenstam (1978) to constitute the Afromontane pachycaul taxa. *Dendrosenecio* was initially classified in *Senecio* based on the striking similarities in floral characters. It is therefore not surprising that the elevation of *Dendrosenecio* was at first controversial (Jeffrey et al. 1977) as the genus exhibited substantial morphological resemblances to other African perennials of *Senecio*. Despite these remarkable morphological similarities, amplified fragment length polymorphism analysis revealed considerable divergence between *Senecio* and *Dendrosenecio* (Knox and Palmer 1995b). Afterwards, internal transcribed spacer (ITS) data identified *Oresbia* Cron & B. Nord. as the closely related genus to *Dendrosenecio* (Pelser et al. 2007).

Majority of the segregated groups are now accepted on the basis of molecular data obtained from markers such as ITS (Pelser et al. 2007). However, it is evident that more valuable diagnostic molecular sequences are needed to further understand the generic and intergeneric relationships in Senecioneae. The large number of species, considerable variation in species life-history and over-dependence on morphological characters, the majority of which overlap, have been pointed out as the causes of the systematic conflict observed within *Senecio*. Similar to *Senecio*, infrageneric relationships within *Dendrosenecio* are still debatable, especially in relation to specific and subspecific classifications. Species of *Dendrosenecio* exhibit ‘mosaic of morphological variation’ arising from divergence and convergence as they dispersed to various geographical regions with similar habitat conditions (Knox 2005; Mabberley 1973). Besides, frequent hybridization events between species within each genus have been evidenced resulting in allopolyploid species (Hedberg 1957; Hegarty et al. 2012; Milton 2009). It is therefore imperative that more molecular markers and divergent regions are identified to facilitate species identification, speciation and adaptive evolution studies on species of *Senecio* and *Dendrosenecio*.

Partial plastid markers, species-specific or universal, have in the past decades been used to resolve phylogenetic relationships and species delimitations. This inclination is progressively being substituted by the use of plastid genome-scale data, resulting in improved phylogenetic resolutions and detailed evolutionary information about species at all taxonomic levels. Typically chloroplast DNA is uniparentally, maternally in angiosperms and paternally in gymnosperms, inherited and exhibits homologous recombination (Marechal and Brisson 2010). This attribute can greatly benefit studies on taxa that are affected by hybridization, introgression and convergent evolution. Additionally, chloroplast genomes are justifiably conserved in terms of gene composition and arrangement permitting comparative genomics even at the generic level. However, they harbour key variations e.g., in the inverted repeat (IR) size and positioning of the IR junctions even among close relatives (Downie and Jansen 2015), and in specific lineages, massive rearrangements, gene duplications, loss or gain have been observed e.g. in Campanulaceae (Knox 2014). These variations provide sufficient unique attributes to reconstruct phylogenetic relationships with strong statistical support, and to investigate the origin and evolutionary patterns of plastids (Pouchon et al. 2018; Tonti-Filippini et al. 2017) through comparative genomics.

To date, only eight chloroplast genomes have been sequenced and reported from three genera in the Senecioneae tribe (Doorduin et al. 2011; Lee et al. 2016; Wang et al. 2019). In this study, the first five and six chloroplast genomes in *Senecio* and *Dendrosenecio* respectively were sequenced and analysed. The objectives were: to generate, characterize and analyse the complete chloroplast genomes of 11 species of Senecioneae; to identify highly variable regions that could be of phylogenetic utility within the tribe through comparative analyses and; to investigate the potential of chloroplast phylogenomics in resolving phylogenetic relationships among the species of Senecioneae, with key interest on the phylogenesis of *Senecio* and *Dendrosenecio*.

## Methods

### Plant material and genome sequencing

Fresh young leaves of 11 species of Senecioneae were collected from the tropical mountains in eastern Africa (Table 1). The species were identified according to the morphological descriptions given in the Flora of Tropical East Africa (Beentje et al. 2005; Knox 2005). Voucher specimen for each species was deposited in the Herbarium of Wuhan Botanical Garden, Chinese Academy of Sciences (HIB). Total genomic DNA was extracted from approximately 100 mg of leaf material for each sample using a modified 2 × cetyltrimethylammonium bromide (CTAB) method (Doyle 1987). The DNA quality was checked using Qubit 2.0 Fluorometer (Life Technologies, CA, USA). A DNA library was constructed for each species by shearing the genomic DNA into short fragments of ~ 350 bp. The DNA was sequenced based on the pair-end sequencing technique implemented on an Illumina Hiseq 2500™ platform (Illumina Inc., San Diego, CA, USA). An average of 22.75 million paired reads, at least 5 Gb of raw sequence data, were generated for each species.

**Table 1:** Characteristics of complete chloroplast genomes of 14 species of the tribe Senecioneae (Asteraceae)

General genome characteristic	Source	Herbarium accession numbers	GenBank accession number	Genome size (bp) [GC%]	LSC length (bp)	SSC length (bp)	IR length (bp)	PCGs (duplicated in the IR)	Trnas (duplicated in the IR)	Rrna (duplicated in the IR)
*Dendrosenecio johnstonii* (Oliv.) B. Nord.	Mt. Kilimanjaro	SAJIT-002716	MG560050	150,607 [37.5]	83,469	17,754	24,692	80 (8)	30 (7)	4 (4)
*D. meruensis* (Cotton & Blakelock) E.B. Knox	Mt. Meru	SAJIT-002556	MG560049	150,587 [37.5]	83,448	17,755	24,692	80 (8)	30 (7)	4 (4)
*D. elgonensis* subsp. *elgonensis* (T.C.E.Fr.) E.B. Knox	Mt. Elgon	SAJIT-003220	KY434194	150,548 [37.5]	83,403	17,771	24,687	80 (8)	30 (7)	4 (4)
*D. keniodendron* (R.E. Fr. & T.C.E.Fr) B. Nord.	Mt. Kenya	SAJIT-002100	KY434193	150,555 [37.5]	83,418	17,755	24,691	80 (8)	30 (7)	4 (4)
*D. battiscombei* (R.E. Fr. & T.C.E.Fr) E.B. Knox	Mt. Kenya	SAJIT-002802	KY434195	150,556 [37.5]	83,410	17,762	24,692	80 (8)	30 (7)	4 (4)
*D. brassiciformis* (R.E.Fr. & T.C.E.Fr) Mabb.	Aberdares Ranges	SAJIT-003289	MG560051	150,551 [37.5]	83,424	17,747	24,690	80 (8)	30 (7)	4 (4)
*Senecio moorei* R.E.Fr.	Mt. Kenya	SAJIT-201834	MH483949	151,204 [37.2]	83,278	18,300	24,813	80 (8)	30 (7)	4 (4)
*S. keniophytum* R.E.Fr.	Mt. Kenya	SAJIT-201831	MH483946	151,413 [37.2]	83,422	18,277	24,857	80 (8)	30 (7)	4 (4)
*S. purtschelleri* Engl.	Mt. Kenya	SAJIT-201832	MH483947	151,191 [37.2]	83,243	18,302	24,823	80 (8)	30 (7)	4 (4)
*S. schweinfurthii* O.Hoffm.	Mt. Kenya	SAJIT-201833	MH483950	151,260 [37.2]	83,255	18,389	24,823	80 (8)	30 (7)	4 (4)
*S. roseiflorus* R.E.Fr.	Mt. Kenya	SAJIT-201835	MH483948	151,228 [37.2]	83,329	18,279	24,810	80 (8)	30 (7)	4 (4)
*Jacobea vulgaris* Gaertn.	–	–	NC_015543	150,689 [37.3]	82,855	18,277	24,777	81 (8)	29 (7)	4 (4)
*Ligularia fischeri* (Ladeb.) Turcz.	–	–	KT988070	151,133 [37.5]	83,238	18,233	24,831	80 (8)	29 (7)	4 (4)
*Pericallis hybrida* (Regel) B.Nord	–	–	NC_031898	151,267 [37.3]	85,751	18,331	23,591	79 (8)	30 (7)	4 (4)

### Genome assembly and annotation

The raw data were filtered and trimmed using Fastp software using the default settings (Chen et al. 2018); all low-quality reads were discarded. The de novo assembly of the filtered reads, into complete chloroplast genomes, was performed using NOVOPlasty (Dierckxsens et al. 2017) with default seed and K-mer = 31–39. The contigs were then mapped to the chloroplast genomes of *Jacobaea vulgaris* Gaertn. (NC_015543; Doorduin et al. 2011) and *Pericallis hybrida* (Regel) B. Nord. (NC_031898; Wang et al. 2019) using Geneious Prime 2019 (Biomatters Ltd., Auckland, New Zealand; https://www.geneious.com). Basic local alignment search tool ver. 2.2.18+ (Camacho et al. 2009) was used to ascertain the positions of the single copies and the inverted repeat regions by self-blasting the assembled sequences.

GeSeq (Tillich et al. 2017) was used to annotate each of the chloroplast genomes using the complete chloroplast genome sequences of *Jacobaea vulgaris* and *Pericallis hybrida* as references. Where necessary, manual corrections were performed in Geneious Prime 2019 (Biomatters Ltd., Auckland, New Zealand), to rectify the start and stop codons of the protein-coding genes (PCGs), based on the annotations of *J. vulgaris* and *P. hybrida*. A circular genome map for each species was generated using OGDraw v1.2 (Lohse et al. 2007). All annotated genome sequences were submitted to the GenBank under the accession numbers listed in Table 1.

### Codon usage and microsatellite repeats identification

The level of codon usage bias was determined by analysing the Relative Synonymous Codon Usage (RSCU; Sharp and Li 1987), Effective Number of codon (ENc; Wright 1990) and the Codon Biased Index (CBI; Morton 1993) for all PCGs, in DnaSP 6.10 (Rozas et al. 2017). The frequency of amino acid was also considered. The MicroSAtellite Identification tool Perl Script (MiSa; Thiel et al. 2003), was used to mine for SSRs with the parameters set at 10 for mononucleotides, 5 for dinucleotides, 4 for trinucleotides and 3 for tetra-, penta- and hexa-nucleotides.

### Genome comparative analyses and divergence hotspot identification

The available chloroplast genomes of Asteraceae species have been shown to harbour no major differences in their sizes, gene content and arrangement. The whole genome size, GC percentage, LSC, SSC, IR and number of gene in each of the 11 species, were therefore compared to three other species of Senecioneae. Preliminary comparative analyses among the species within each genus revealed highly conserved sequences with > 99% pairwise identity and > 98% identical sites. Consequently, one chloroplast genome sequence was randomly picked per genus to conduct further comparative studies against other chloroplast genomes within the tribe. The expansion/contraction of the IR regions was assessed by comparing the positions of SC/IR junctions and their adjacent genes using IRscope (Amiryousefi et al. 2018).

Further, to outline any significant sequence divergence spots and genome rearrangements, the chloroplast genomes were aligned and plotted in MAUVE (Darling et al. 2004), with *Nicotiana tabacum* L. (NC_001879; Shinozaki et al. 1986) being used as an external reference genome. Nucleotide diversity (Pi) in the non-coding regions (> 200 bp) of the five species of Senecioneae was analysed in DnaSP v.6.10 (Rozas et al. 2017). Potential primers for ten sites with the highest Pi values were designed using Primer3 (Untergasser et al. 2012) using default settings.

### Phylogenetic analyses

A total of 75 species, representing 49 genera from 11 tribes of Asteraceae, were downloaded from the NCBI (Additional file 1: Table S1) for phylogenetic analyses. Besides, data for *Adenophora divaricata* Franch. & Sav. and *A. stricta* Miq. (Cheon et al. 2017) were downloaded and used as outgroups in this analysis. Sequences of 70 common PCGs were extracted from the 77 species. Each gene was separately aligned using MUSCLE (Edgar 2004) and then concatenated in Geneious Prime 2019 (Biomatters Ltd., Auckland, New Zealand). Phylogenetic reconstructions were carried out using Maximum Likelihood (ML) and Bayesian Inference (BI) methods. Each method was used twice in independent analyses based on unpartitioned and partitioned data. Before the ML analysis using unpartitioned data, the best-fit DNA substitution model was determined using ModelFinder (Kalyaanamoorthy et al. 2017) as implemented in IQ-TREE version 1.5.4 (Nguyen et al. 2015). Maximum Likelihood (ML) analysis was conducted using IQ-TREE with a bootstrap analysis of 5000 replications under the GTR + F + R6 nucleotide substitution model. MrBayes v3.2.6 (Ronquist et al. 2012) was used to implement the BI analyses based on the unpartitioned data set, using four independent Markov Chain Monte Carlo runs with three heated and one cold chain. The chains were run for 2 × 10^6^ generations with sampling from the cold chain run after every 10^3^ generations. The analysis was stopped after the average standard deviation of split frequencies as calculated by Mr. Bayes was below 0.01, an indication that convergence had been attained. The first 25% of all generations were excluded, and a consensus phylogenetic tree was obtained based on majority rule from the remaining trees. Branch support was indicated by posterior probability (PP) values. The data set was then partitioned by categorizing the nucleotides in each gene based on the position (first, second, or third) they occupy in a codon. The best partitioning scheme and substitution models were calculated using PartitionFinder2 (Lanfear et al. 2017). The generated phylogenetic trees were visualised and formatted in Interactive tree of life (iTOL) v3 (Letunic and Bork 2016).

## Results

### Chloroplast genome organization and content

**Fig. 1:**
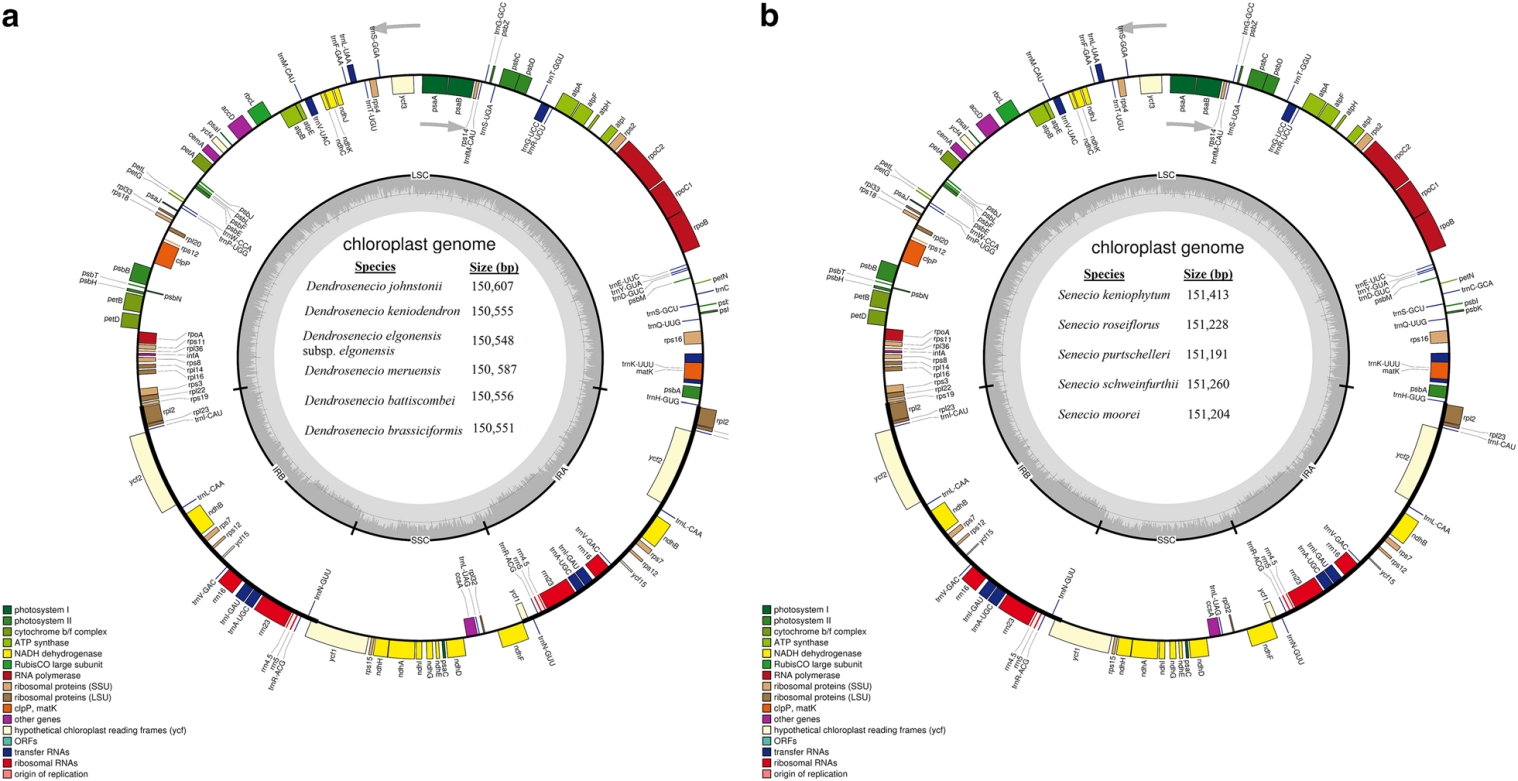
A representative chloroplast genome map of **a** *Dendrosenecio* and **b** *Senecio*. Genes are color-coded based on their function as shown in the legend. The inner circle indicates the inverted repeat boundaries and the genome’s GC content. The arrows indicate the direction of gene transcription

An average of 22.6 million (95.2%) clean reads were generated for each species. The chloroplast genomes of the two genera were comparable in terms of structural organization, gene content, and arrangement. The overall chloroplast genome size varied slightly within each genus, but significantly between the genera ranging within 150 kb in *Dendrosenecio* and to 151 kb in *Senecio*. Each of the chloroplast genomes had four regions including a large single copy of ~ 83.5 kb in both genera, two inverted repeats ~ 24.7 kb, and a small single copy ~ 17 kb in *Dendrosenecio* and ~ 18 kb in *Senecio*. The GC percentage values of the entire genome and for each of the sections were identical in all species within a respective genus (Table 1). Each of the plastid genomes encoded a total of 114 unique genes of which 80 were PCGs, 30 tRNAs and four rRNAs. All the PCGs, except three, had the standard AUG start codon (Table 2). Seventeen genes; 11 PCGs and six tRNA genes contained either one or two introns. Eighteen genes were duplicated in the IR regions, with *rps12* being uniquely positioned with its 5′ end exon at the LSC and the other is located in the IR regions. Both *ycf*1 and *rps*19 genes also had their 3′ ends duplicated on the IR regions (Fig. 1; Table 1).

**Table 2:** List of genes identified in the studied chloroplast genomes of 11 species of Senecioneae

Gene family	Gene
Transfer RNA	*trnA*-UGC*, trnfM-*CAU, trnI*-GAU*, trnM-*CAU, trnR*-*ACG, trnS*-*UGA, trnC*-*GCA, trnG*-*GC, trnK*-UUU*, trnN-*GUU, trnW*-*CCA, trnT*-*GGU, trnD*-*GUC, trnV*-UAC*, trnL-*CAA, trnY*-*GUA, trnR*-*UCU, trnT*-*UGU, trnE*-*UUC, trnH*-*GUG, trnL*-UAA*, trnP-*UGG, trnS*-*GCU, trnV*-*GAC, trnF*-*GAA, trnI*-*CAU, trnL*-*UAG, trnQ*-*UUG, trnS*-*GGA, trnG*-UCC*
Small ribosomal units	*rps2,3,4,7,8,11,12*^*a*^,14,15,16*,18,19^*b*^
Large ribosomal units	rpl2*,14,16*,20,22,23,32,33,36
RNA polymerase sub-units	rpoA, rpoB, rpoC1*, rpoC2
Translation initiation facto	*infA*
NADH dehydrogenase	ndhA*, ndhB*, ndhC, ndhD^*b,*^ *ndhE, ndhF, ndhG, ndhH, ndhI, ndhJ, ndhK*
Photosystem I	psaA, psaB, psaC, psaI, psaJ, ycf3**, ycf4
Photosystem II	*psbA,B,C,D,E,F,H,I,J,K,L*^*b*^*,M,N,T,Z*
Cytochrome b/f complex	petA, petB*, petD*, petG, petL, petN
ATP synthase	atpA, atpB, atpE, atpF*, atpH, atpI
Large subunit of rubisco	*rbcL*
Maturase	*matK*
Protease	clpP**
Acetyl-CoA-carboxylase sub-unit	*accD*
Envelope membrane protein	*cemA*
Component of TIC complex	*ycf1*
c-Type cytochrome synthesis	*ccsA*
Hypothetical genes reading frames	*ycf2, ycf15*
Ribosomal RNA	*rrn4.5, rrn5, rrn16, rrn23*

* Genes with a single intron, ** genes with two introns, ^a^ trans-spliced genes, ^b^ genes with an alternative start codon

### Codon usage and microsatellite sequences

**Fig. 2:**
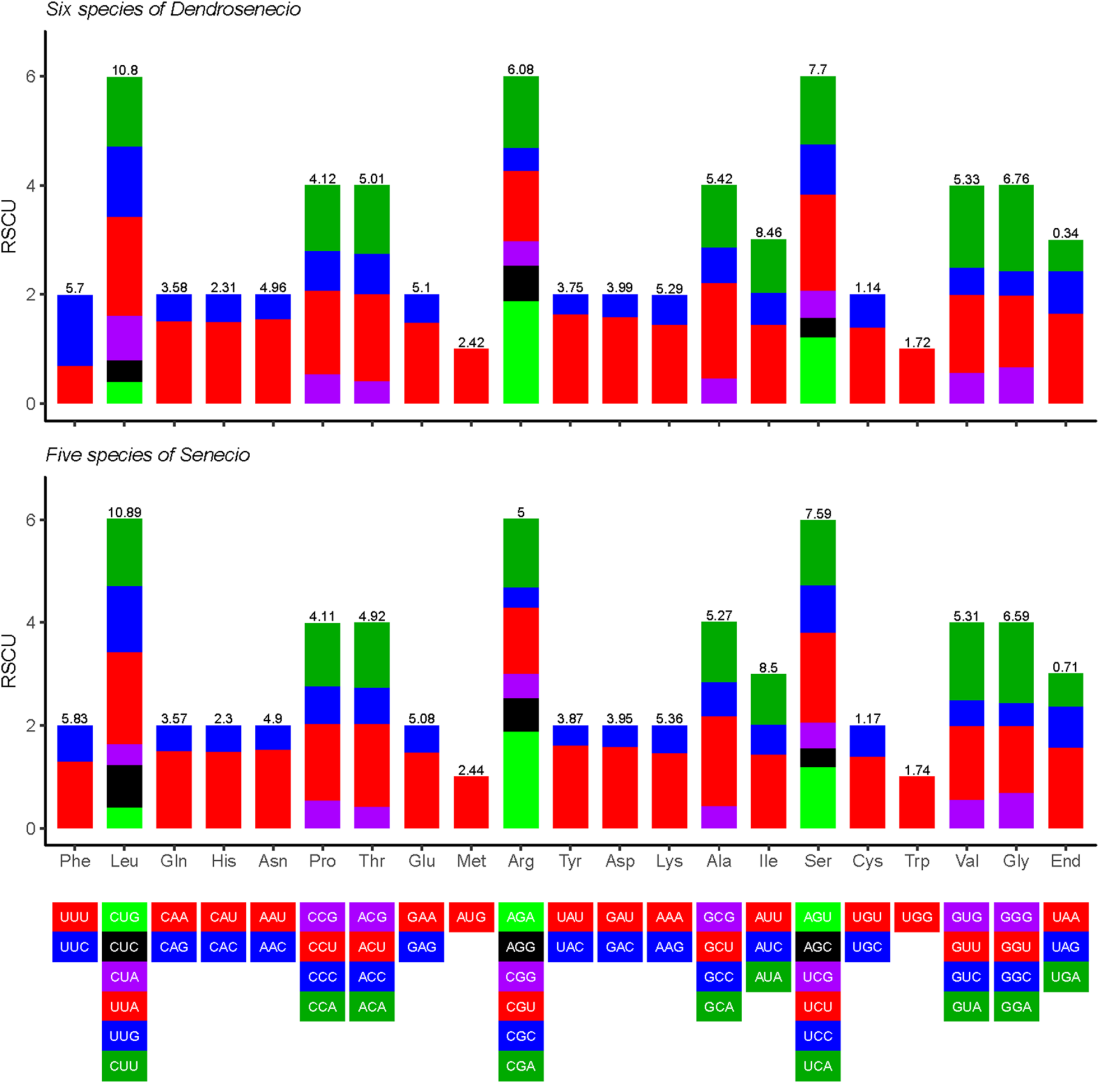
Details of codon usage biasness in the chloroplast genomes of five species of *Senecio* and six species of *Dendrosenecio.* The values at the top of each stack indicate the usage frequency of each amino acid, while the bars (colour coded) depicts the relative synonymous codon usage values for each codon

The total sequence size coding for protein genes was 78,879–79,146 bp in *Dendrosenecio* and 78,203–78,531 in *Senecio.* These protein sequences encoded 26,293–26,382 and 26,067–26,177 codons respectively, including stop codons. Leucine was encoded by the highest number (average = 10.81% and 10.9%) of codons, whereas cysteine (average = 1.14% and 1.13%) was the least encoded in *Dendrosenecio* and *Senecio* respectively. Except for Methionine (AUG) and Tryptophan (UGG), whose RSCU values were 1 in all species, the usage of the other codons was biased. Generally, the usage of seven codons, eight in *Senecio*, was overrepresented (RSCU > 1.6) while the majority had low representation RSCU < 0.6. The ENc ranged from 49.76 to 51.49, while CBI ranged between 0.308 and 0.356 (Additional file 2: Table S2). The average RSCU and amino acid frequency values for each species were plotted using R-script (Fig. 2; Zhang et al. 2018).

**Fig. 3:**
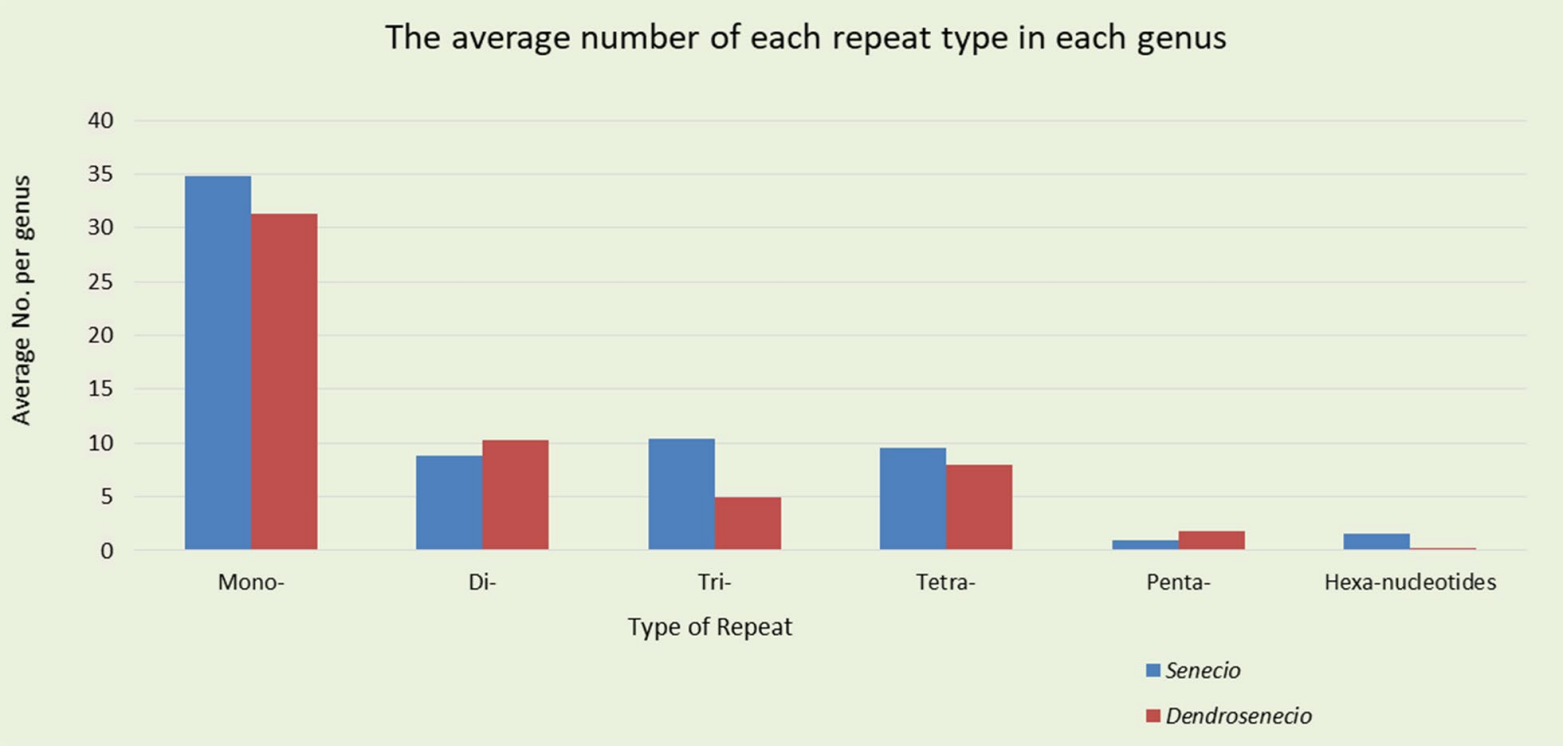
The average number of each type of microsatellites in the chloroplast genomes of *Dendrosenecio* and *Senecio*

In each genus, the analyses of repetitive sequences revealed minimal variation in the total number and position of each repeat motif, most of which were shared among the chloroplast genome sequences. In *Dendrosenecio*, 340 microsatellites were discovered while the species of *Senecio* had 331 SSR repeats. On average, species of *Senecio* had the highest number of mono-, tri-, tetra- and hexa-nucleotides, while di- and penta-nucleotides were the most abundant repeats in *Dendrosenecio* (Fig. 3). Certain repeat motifs were genus-specific while a few e.g., C/G and AGCTAT/AGCTAT in *D. johnstonii* and AATCT/AGATT and AATTC/AATTG in *S. keniophytum* were species-specific (Additional file 3: Table S3). The present SSRs were classified based on the variation in repeat type, the number of repeats in each motif, presence or absence of the repeat, and the position of each repeat in the genome. Microsatellites were considered polymorphic if they: showed variation, were present in all plastid genomes and were positioned at homologous regions across all species in each genus. Based on this criterion, 25 polymorphic SSRs were discovered in *Senecio* and only five in *Dendrosenecio* (Tables 3, 4).

**Table 3:** Details of 25 potentially polymorphic microsatellite repeats in five species of *Senecio*

Microsatellites	*Senecio schweinfurthii*		*S. keniophytum*		*S. moorei*		*S. purtschelleri*		*S. roseiflorus*		Location
	Start	End	Start	End	Start	End	Start	End	Start	End	
(AAAT)3;(ATT)4	1849	1860	1881	1892	1860	1871	1848	1859	1881	1892	Intron
(T)10,11;(T)16n(TTAGA)3	8685	8694	9134	9180	8697	8706	8689	8699	8317	8326	IGS
(T)10;(A)10;(A)10tca(T)10	9738	9747	9720	9729	9738	9760	9731	9754	9695	9704	IGS
(T)17,10,16,	10,859	10,875	10,857	10,866	10,872	10,887	10,866	10,881	10,852	10,861	IGS
(T)11,23,16	12,933	12,943	12,919	12,941	12,945	12,955	12,919	12,929	12,925	12,940	IGS
(T)16,16,15	16,878	16,893	18,219	18,228	16,890	16,905	16,864	16,878	16,875	16,884	Intron
(AT)6;(A)10(TA)6	26,849	26,860	26,787	26,798	26,861	26,872	26,834	26,845	26,776	26,797	IGS
(T)10 g(A)11;(T)10c(A)10;(A)14	28,219	28,240	28,166	28179	28,231	28,252	28,204	28,225	28,154	28,174	IGS
(ATAAA)3;(T)10;(CTA)5	32,007	32,021	30,007	30,016	32,019	32,033	31,992	32,006	31,008	31,022	IGS
(AT)5,7;9	34,922	34,931	34,890	34,916	34,934	34,943	34,907	34,916	34,879	34,892	IGS
(A)11;(T)14	41,864	41,874	41,844	41,857	41,876	41,886	41,849	41,858	41,840	41,849	IGS
(A)17,14,20	46,644	46,660	46,666	46,679	46,656	46,672	46,629	46,645	46,621	46,640	IGS
(T)10,11,12;(A)10n(T)11	48,099	48,109	48,125	48,136	48,111	48,120	48,084	48,093	48,063	48,103	IGS
(A)11.12	59,324	59,334	59,388	59,398	59,335	59,346	59,308	59,318	59,021	59031	IGS
(A)12	64,609	64,620	64,721	64,732	64,621	64,632	64,593	64,605	64,658	64,668	IGS
(A)10;(ATTT)3n;(A)11n(A)11	70,356	70,365	70,409	70,428	70,368	70,377	70,343	70,352	70,266	70,407	Intron
(T)14,13,12	70,577	70,590	70,699	70,710	70,589	70,601	70,564	70,577	70,619	70,630	Intron
(A)11,12,14	76,913	76,923	77,044	77,055	76,925	76,935	76,900	76,910	76,964	76,977	IGS
(T)10,17	77,160	77,169	77,292	77,308	77,172	77,181	77,147	77,156	77,215	77,231	CDS
(T)14n(T)16,17;(T)10	79,597	79,716	79,745	79,754	79,609	79,728	79,584	79,704	79,668	79,677	IGS
(A)14,17	80,181	80,194	80,303	80,319	80,193	80,206	80,169	80,182	80,221	80,237	IGS
(A)10,11,15	108,867	108,877	109,796	109,805	108,880	108,890	108,855	108,865	109,680	109,694	CDS
(A)12,16	110,440	110,451	110,599	110,614	110,453	110,464	110,428	110,439	110,483	110,494	CDS
(GATT)3;(GATT)3n(TAAT)3	115,162	115,173	115,324	115,409	115,174	115,185	115,149	115,160	115,206	115,291	Intron
(AT)5,6	115,559	115,570	115,973	115,982	115,571	115,582	115,546	115,557	115,947	115,957	Intron

n = varying number of base pairs between two repeat motifs

**Table 4:** Details of potentially polymorphic microsatellite repeats in six species of *Dendrosenecio*

Microsatellites	*D. keniodendron*		*D. elgonensis* subsp. *elgonensis*		*D. battiscombei*		*D. meruensis*		*D. johnstonii*		*D. brassiciformis*		Location
	Start	End	Start	End	Start	End	Start	End	Start	End	Start	End	
A13,14,16;TnA29	42,047	42,059	42,044	42,057	42,051	42,064	42,044	42,072	42,053	42068	42,049	54,600	Intron
(T)10,11,13	59,146	59,155	59,130	59,140	59,150	59,160	59,179	59,189	59,200	59,210	59,147	59,210	IGS
(A)10,11,12	70,469	70,478	70,456	70,465	70,474	70,483	70,368	70,378	70,521	70,532	70,473	70,532	IGS
(T)11,12,15	79,748	79,762	79,736	79,746	79,740	79,754	79,781	79,791	79,802	79,813	79,802	79,813	IGS
(A,AgaaatattttttgtA,AgaaatattttttgtA) 11,16,39,40,	80,327	80,342	80,311	80,327	80,319	80,334	80,333	80,372	80,355	80,393	80,355	80,393	IGS

### Genome comparative analyses

**Fig. 4:**
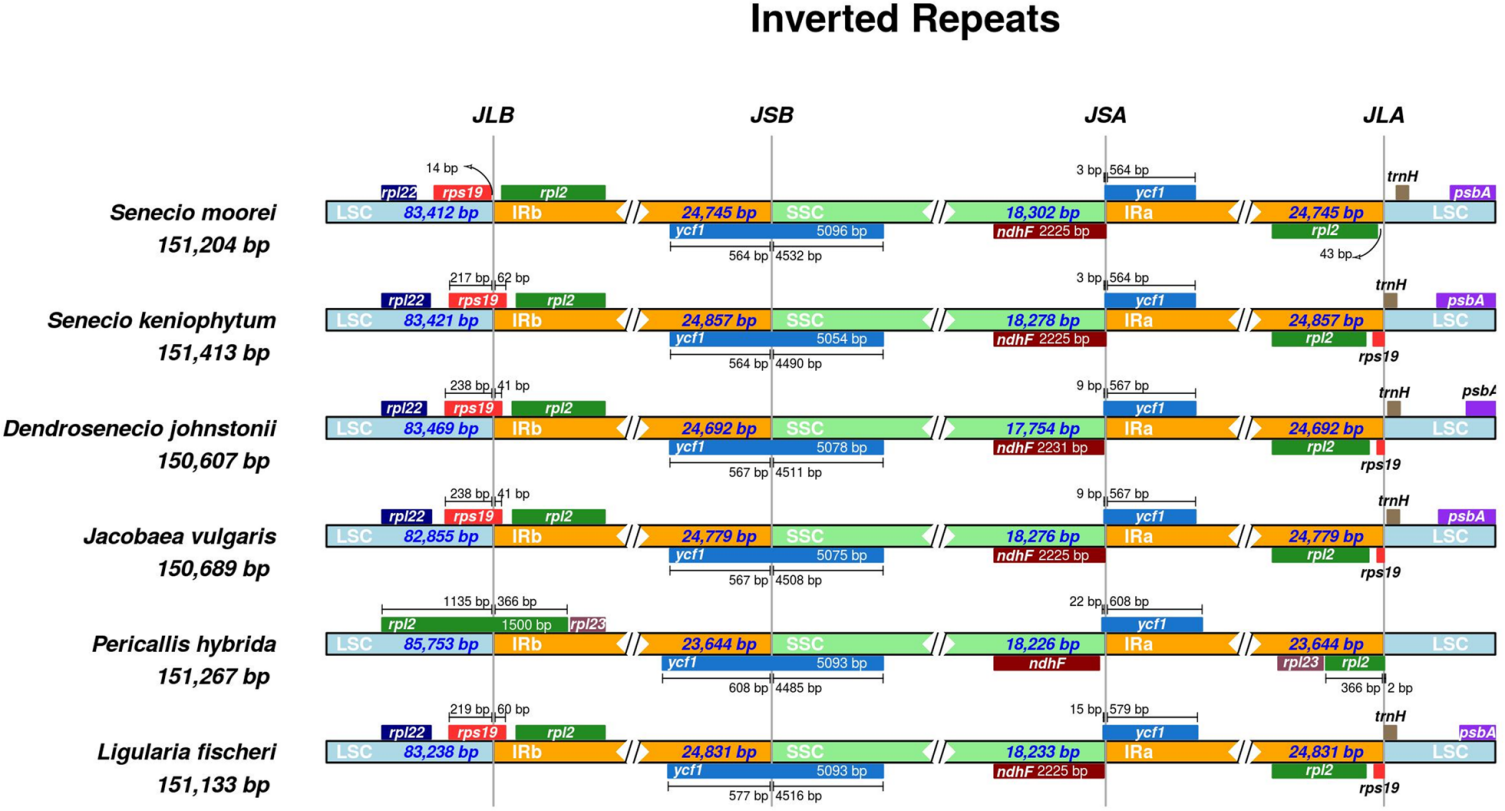
Comparison of the large single copy, inverted repeats, and small single copy junction positions in six species of Senecioneae (Asteraceae). Genes adjacent to the junctions are shown as blocks of different colours

In each genus, the newly annotated chloroplast genomes had no significant differences, except for the slight variations in size and gene positioning. The size difference between the largest and the smallest genome among the *Dendrosenecio*s and *Senecio* was 59 bp and 222 bp respectively. Based on the currently available chloroplast genomes, *Dendrosenecio*s had the smallest chloroplast genome size within the Senecioneae tribe, with a difference of 82 bp from the immediate largest cp genome (*Jacobaea vulgaris*; Table 1). The genes adjacent to the IR/SC junctions (*trnH*, *rps19*, *ycf1* and *ndhF*) were similar in all species, except in *Pericallis hybrida* which had *rpl2* at the LSC/IR junction. The IRb region expanded into the coding region of *rps19,* resulting into a pseudogene (ψ) of varying length at the IRa in all but in *P. hybrida* and *S. moorei*. In *S. moorei*, a 14 bp gap was observed between the *rps19* gene and the JLB border. At the JSB junction, the IRb expanded into the coding region of *ycf1* gene; thus ψ*ycf1* appeared on the IRa region in all species. Two genes, *ndhF* and ψ*ycf1*, were positioned at varying points adjacent to the JSA junction. In *J. vulgaris* and *Ligularia fischeri* (Ladeb.) Turcz., the *ndhF* and ψ*ycf1* genes overlapped. The JLA junction was uniformly flanked between ψ*rps19* and *trnH* genes, except in *S. moorei* and *P. hybrida* where *rpl2* and *trnH* bordered the junction. Figure 4 shows the genes adjacent to the junctions and their order in representative genomes from each genus. In *Senecio* two species were used to show the differences recorded at JLA and JLB in *S. moorei*.

**Fig. 5:**
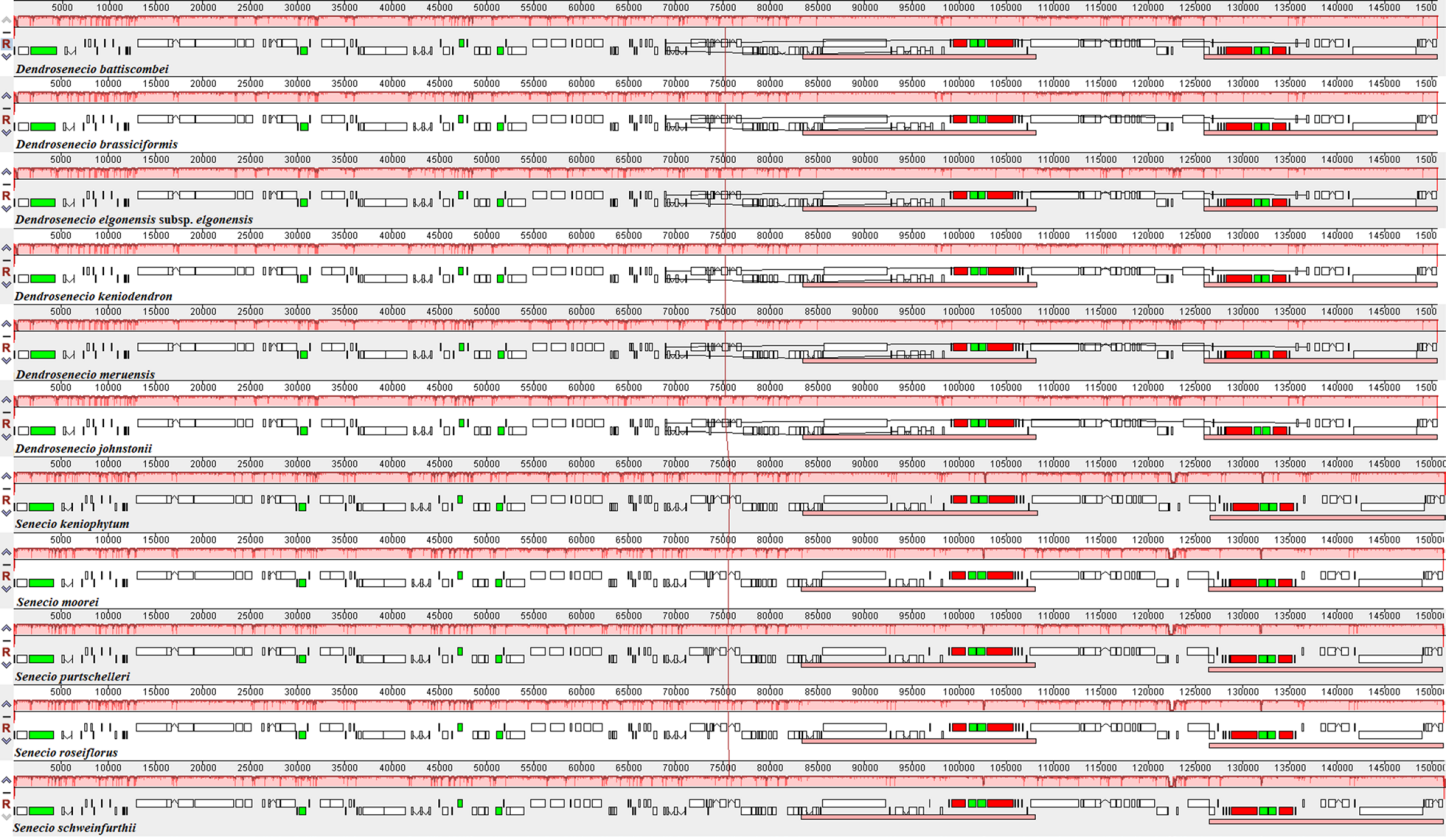
Comparison of sequence arrangement in the chloroplast genomes of 11 species of Senecioneae (Asteraceae). Conserved orthologs are indicated by locally collinear blocks. Similar blocks among the genomes are coded in one colour and joined by a line. The genes above the line are transcribed in a clockwise direction, those below the line are transcribed towards the counter-clockwise direction

**Fig. 6:**
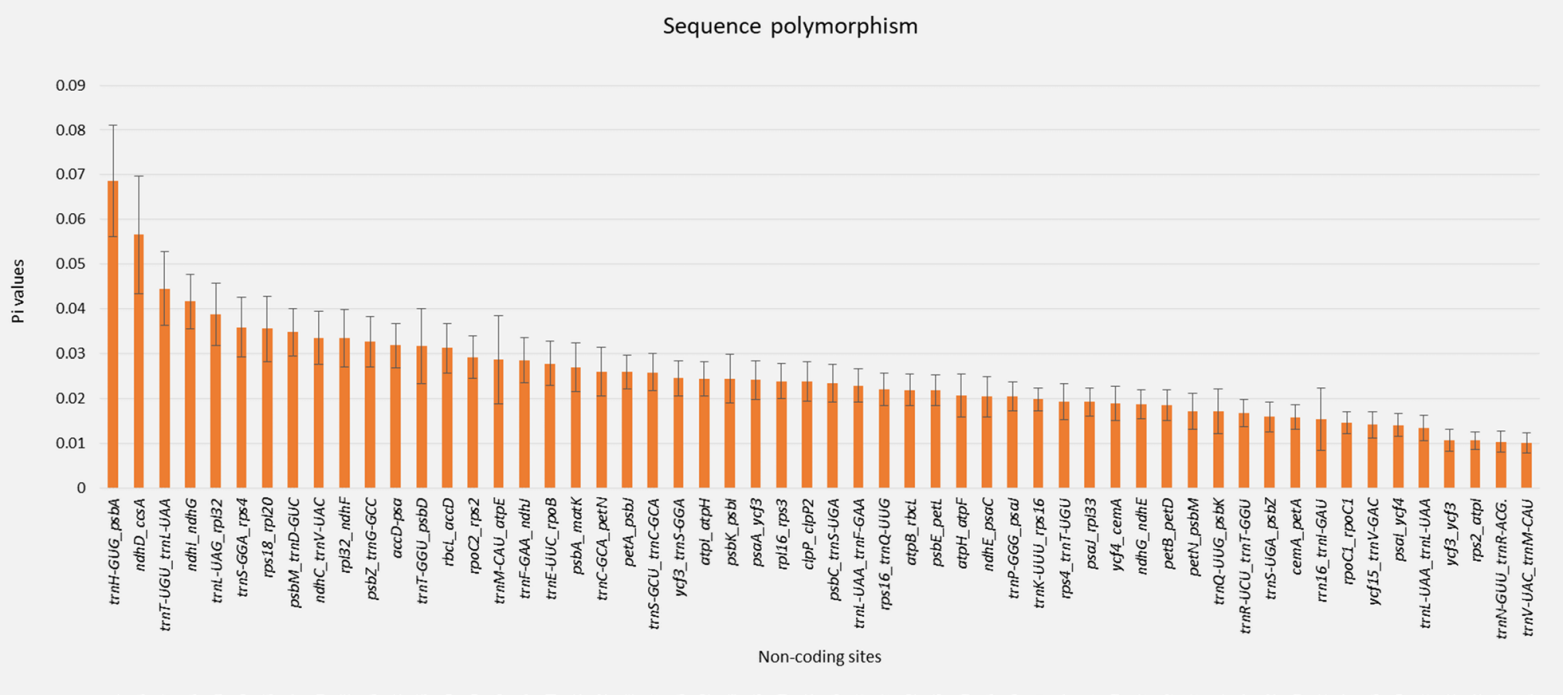
Nucleotide variability (Pi) values of non-coding regions which were extracted from the chloroplast genomes of five species of Senecioneae

There were no major rearrangements detected among the newly sequenced chloroplast genomes, an indication that chloroplast genomes within these two genera could be much conserved (Fig. 5). However, the existence of two inversions in the LSC region in reference to *Nicotiana tabacum* was identified in all newly generated chloroplast genomes. The arrangement of genes in the SSC region was also different in the Asteraceae species, apart from *L. fischeri*, whose alignment was identical to that of *N. tabacum* (Additional file 4: Figure S1). The nucleotide polymorphism test identified 74 sites with Pi values ranging from 0.00089 (*ndhB*–*ndhB*) to 0.06852 (*trnH*-*GUG*-*psbA*). Figure 6 indicates the regions with high levels of intergeneric variation (Pi values > 0.01). Potential PCR primers were designed for the ten highly polymorphic sites (Table 5).

**Table 5:** Details of ten primers that target the most divergent regions in the chloroplast genomes of Senecioneae species

Orientation	Primers	Tm	Target region	Expected product size
F	AAATAGGAGGAAGCTGTGAC	55	*trnH*-*psbA*	603
R	GCACTATGGCTTTCAACCTA	55		
F	CACGTCAGATGTTCTATGGA	55	*ndhD*-*ccsA*	658
R	TTGGGCCTTTATTACTTGGA	55		
F	TTTGCGAAAAGAGGAAGACA	55	*trnT*-*trnL*	812
R	TCCATACCAAGGCTCAATTC	55		
F	TGTGGTAATTGCGTTGAGTA	55	*ndhI*-*ndhG*	759
R	TACGTAAATAAGGAGCTGCG	55		
F	CTTGCTTGTACCTACCCAAT	55	*trnS*-*rps4*	588
R	TGACTCTTCAAGCATTCCAA	54		
F	TTGACCTTGAAACAACAACG	55	*rps18*-*rpl20*	546
R	ACAAGAGACAGTTGCTTCTT	55		
F	GGAACTAAAATGAACAGTGCA	54	*psbM*-*trnD*	880
R	ATGTCTACGCTGGTTCAAAT	55		
F	GCGGATTCTAAATTGTAACCA	54	*ndhC*-*trnV*	959
R	AGCCCTAGAGCCCTATAAAA	55		
F	TAACGCTGCCAAATATCCTT	55	*rpl32*-*ndhF*	1194
R	AGAAGAAGTCCCAACCCTAT	55		
F	ATTGCCACTTCATCAATCTT	53	*psbZ*-*trnG*-	617
R	ACTACACTATGACGGCTAAC	54		

*Tm* annealing temperature, *F* forward primer, *R* reverse primer

### Phylogenetic relationships

**Fig. 7:**
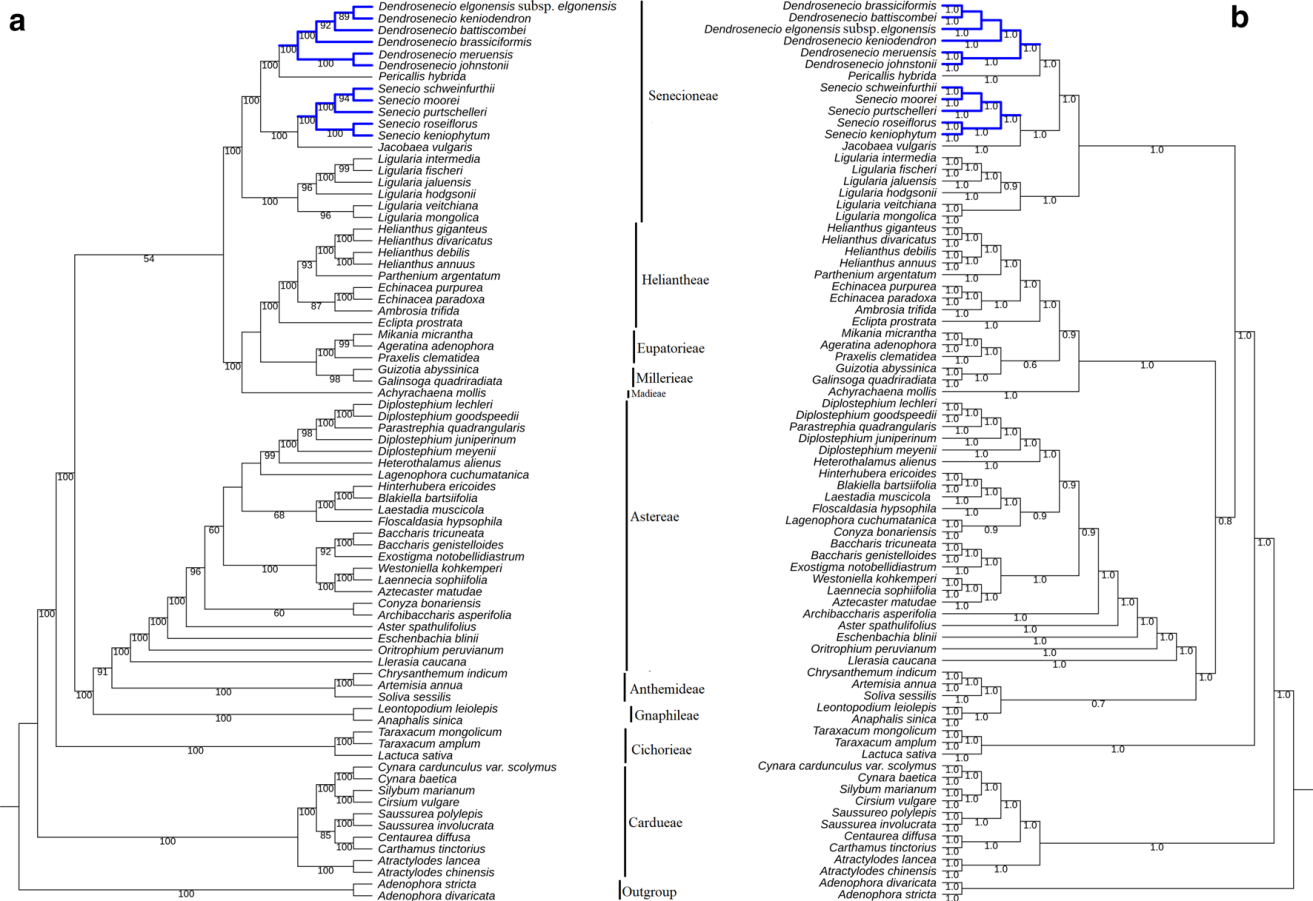
Phylogenetic relationships of 75 species of Asteraceae inferred from an unpartitioned multi-gene dataset using **a** Maximum Likelihood (ML) and **b** Bayesian Inference methods

The final sequence alignment of common protein-coding genes had 60,992 characters in 70 chloroplast genome loci for 77 taxa. Phylogenetic relationships among the 75 species representing ten tribes of Asteraceae were unveiled based on ML and BI analyses. The tribes were recovered as monophyletic clades each with significant statistical support in all the generated trees. Intergeneric relationships within tribe Senecioneae, to which the 11 newly sequenced chloroplast genomes belong, were clearly defined and strongly supported in all data schemes (100% BS and 1.0 PP). The phylogenetic analyses strongly supported three sub-clades within the tribe Senecioneae; one that comprised of the genus *Ligularia*, the second sub-clade contained both *Senecio* and *Jacobaea* while the third one had species from *Dendrosenecio* and *Pericallis*. The sister relationship between the species of *Senecio* was congruent in all the analyses, differing only in support values at the clade containing *S. moorei* and *S. schweinfurthii* which was highly supported (BS ≥ 92) in ML trees but gained weak support in BI analyses (PP ≤ 0.5). The six species of *Dendrosenecio* were split into two clades, distinctly separating species from Tanzania and species from Kenya (Fig. 7). However, the interspecific relationship within the Kenyan species differed in the different phylogenetic trees (Additional file 5: Figure S2).

## Discussion

### Genome structure and content

It is typical of higher plants to possess a chloroplast genome that has a quadripartite structure and a relatively well-conserved gene content and arrangement (Ravi et al. 2008; Wicke et al. 2011; Yurina et al. 2017). However, minor to major variability in chloroplast genome structure has been observed in specific plant lineages (Guisinger et al. 2011). The current upsurge in the amount of chloroplast genome-scale data has played a crucial role in the enhancement of knowledge on the evolution and organization of chloroplast genomes. Both *Senecio* and *Dendrosenecio* are conspicuous genera in the tribe Senecioneae, the former for being the largest genus in the tribe and the latter for exhibiting an atypical growth habit. In this study, the first complete chloroplast genome sequences of five *Senecio* and six *Dendrosenecio* species were generated. The genome structures were comparable to those of higher plants each exhibiting four compartments; a pair of inverted repeats and a pair of single copies of unequal length. The overall chloroplast genome size of between 150 and 151 kb, was comparable to the majority of chloroplast genomes of species within the Asteraceae family (Salih et al. 2017). The GC content of species within each of the two genera was identical 37.2% in *Senecio* and 37.5% in *Dendrosenecio* (Table 1, Fig. 1). Usually, chloroplast genomes exhibit a high AT-GC content ratio (Ravi et al. 2008), which is a crucial factor in genome organization and stability (Niu et al. 2017).

Chloroplasts have undergone enormous changes since they evolved from cyanobacteria in over a billion years ago (Timmis et al. 2004). One of the notable transformations is the reduction in size, which is primarily accredited to considerable gene transfer from the chloroplast genome to the nuclear genome (Bock and Timmis 2008; Martin et al. 1998). Generally, the chloroplast genomes of higher plants currently encode 70–90 protein-coding genes, which is approximately 2% of the total PCGs found in cyanobacterium *Synechocystis* (Eckardt 2006). The number of unique genes encoded in the available chloroplast genomes of Asteraceae varies slightly between 110 and 115 (Dempewolf et al. 2010; Doorduin et al. 2011; Kumar et al. 2009; Lu et al. 2016; Walker et al. 2014). The 11 species reported here encoded an equal number of 114 unique genes, with 80 genes encoding for protein, similar to the chloroplast genomes of *Taraxacum* F.H. Wigg. (Asteraceae; Salih et al. 2017). The exon of some specific genes has been subjected to interruptions by either a single or several introns. In such cases, the entire sequence, containing both the exon and intron(s), is transcribed into a forerunner RNA and later the introns are detached for accurate production of a proper transcript (Eckardt 2007; Plant and Gray 1988). The *rps12* gene is distinctively placed, with its 5′-end being positioned in the LSC region and the 3′-end is duplicated in the IR section, in a way similar to that of most other angiosperms including *Nicotiana tabacum* (Hildebrand et al. 1988). Seventeen genes, in each of the studied species, had either a single or several introns (Table 2). Excluding *rps12* gene whose intron is exceptionally large, *trnK*-*UUU* had the largest single intron. Two genes, *ycf3* and *clpP,* had two introns within their exons this arrangement has also been observed in other Asteraceae species including *Artemisia annua* L. (Shen et al. 2017). However, in *Ageratina adenophora* (Spreng.) R.M. King. & H. Rob., the *rpoC1* gene has two introns (Nie et al. 2012), which is considered rare among other Asteraceae species.

### Codon usage and repetitive sequences

Relative synonymous codon usage values of less than one indicate that the codons are less frequent, > 1 represents those that are more frequently used, whereas = 1 shows no bias (Uddin 2017). An identical trend in the manner in which the amino acids were encoded was discovered among the 11 species (Fig. 2, Additional file 2: Table S2). The usage frequency of leucine was higher than the rest, while cysteine had the least frequency which is congruent to most Asteraceae species e.g., (Salih et al. 2017; Shen et al. 2017). It was also observed that usage of synonymous codons was generally biased in favour of those ending with A/U bases. Consequently, some codons were over- (> 1.6) or under- (< 0.6) represented. In particular, only *trnL*-*UAA*, *trnS*-*UCU*, *trnT*-*ACU*, *trnA*-*GCU, trnY*-*UAU, trnR*-*AGA*, and stop codon UAA were over-represented, whereas the majority were under-represented (Additional file 2: Table S2). Methionine (AUG) and tryptophan (UGG) were uniformly used (RSCU = 1). Other indices of non-uniformity in codon usage include the Effective Number of codons (ENc), which ranges from 20 (one codon per amino acid) to 61 (equal use of synonymous codons; Wright 1990) and Codon Bias Index (CBI) which ranges from 0, no bias to 1 equal usage of all the synonymous codon (Morton 1993). The values for both ENc (49.76 to 51.49) and CBI (0.308 to 0.356) were insignificantly different among the species of *Dendrosenecio,* but similar to most species in Asteraceae (Nie et al. 2014). The common start codon for the protein coding genes is AUG (M) however, three genes, *psbL, rps19,* and *ndhD* deviated from the norm, and they had ACG, GUG, and GUG respectively.

Microsatellite repeats are abundantly distributed in the genome (Tautz and Renz 1984), and they display a high level of polymorphism, placing them among the most preferred genetic markers for genetic investigations. A total of 340 and 331 SSRs were discovered in six species of *Dendrosenecio* and five *Senecio* species. The majority were mononucleotides, followed by dinucleotides and tetranucleotides (Additional file 3: Table S3). Mononucleotides, usually A/T repeat types, are abundantly present in chloroplast genomes of Asteraceae species, e.g., *Jacobaea vulgaris* (Doorduin et al. 2011) *Artemisia annua* (Shen et al. 2017) and other families e.g., *Paeonia ostii* T. Hong & Z. X. Zhang (Paeoniaceae; Guo et al. 2018). In *Dendrosenecio*, a majority of the SSRs were located on homologous regions and except for five, the rest lacked any variations in terms of length and motif. On the contrary, 25 microsatellites in *Senecio* exhibited slight variations based on the same criteria. Being considered polymorphic, the identified microsatellites are therefore potential molecular markers for use in further studies within the respective genus.

### Chloroplast genome comparison

There were no remarkable structural rearrangements among the taxa of these two genera. The chloroplast genomes are highly conserved with an identical structure and an equal number of genes, an indication that this could be the case in chloroplast genomes of most species of these genera. Comparative analyses against representatives of three other genera of Senecioneae revealed a similar trend of the well-preserved structure and organization. The inverted repeat region is present in a majority of angiosperms chloroplast genomes. Initially the IR was reported to serve as a whole-genome stabilizer by reducing recombination between the two SC regions; however, these reports lacked support as more chloroplast genomes revealed significant rearrangements even with both copies of the IR present (Jansen and Ruhlman 2012). Comparative analyses between plants of different plant lineages revealed that inverted repeats could contract/expand up to a few hundred base pairs even among closely related species (Goulding et al. 1996). In this study, the comparison of the IR/SC junctions showed a slight expansion of the IR in all except in *Senecio moorei* and *Pericallis hybrida*. In the novel chloroplast genomes, the same genes were found adjacent to the junctions, and only slight length variations were recorded in *P. hybrida and S. moorei*. In *P. hybrida* the LSC/IRb junction contracted into the *rpl2* gene, whereas it extended into the *rps19* in all the other analysed species. Two pseudogenes (ψ*rps19* and ψ*ycf1)* of varying length were generated in the IRa region, as a result of the expansion of the IR into the exons of *rps19* and *ycf1* genes respectively (Fig. 4). This pattern of expansion of the IR, and the introduction of partial copies of genes with non-coding abilities represents a familiar phenomenon in majority of Asteraceae species (Wang et al. 2015), and besides being a source of DNA barcodes, it can offer insights into the evolutionary processes of plastid genomes.

The entire sequences of the 11 chloroplast genomes generated here, lack any striking inversions or rearrangements and therefore were outlined as a single locally collinear block in our analyses. However, certain regions harboured divergent sites the majority of which were in the non-coding regions. Among the few sites with significant deviations include *trnH*-*(GUG)*-*psbA, ndhD*-*ccsA, trnT(UGU)*-*trnL(UAA), ndhI*-*ndhG,* and *trnL*-*UAG*-*rpl32*. Other regions including *trnL(UAG)*-*rpl32* and the exons of *ndhF* and *ycf1* were within a conserved block, but they had significant divergent points. These findings were supported by results obtained from the DNA polymorphism test based on genus representatives, as same regions were noted to have high nucleotide variability (Pi). Some of these regions have previously been reported in chloroplast genomes of other species (Salih et al. 2017; Wu et al. 2018) and used in phylogenetic studies of numerous taxa including *Senecio* (Kandziora et al. 2016). Non-coding regions in chloroplast genomes have shown high potential for use as molecular markers for phylogenetic studies at low taxonomic levels in Angiosperms (Shaw et al. 2005). Therefore, the regions identified herein are prospective sources of highly informative markers for phylogenetic utility in elucidating intergeneric relationships within the tribe. Subsequently, ten potential markers were developed, allowing for specific amplification of each of the ten most polymorphic sites.

We compared the Senecioneae chloroplast genomes against *Nicotiana tabacum* and detected the two inversions reported to be shared by all clades of Asteraceae family, apart from species of the Barnadesioideae subfamily (Kim et al. 2005). Six conserved gene blocks were identified among the chloroplast genomes indicating the most conserved regions of the genomes. The SSC region in *Ligularia fischeri* was differently oriented in relation to the other Asteraceae species. This re-inversion is considered an ordinary phenomenon among chloroplast genomes of higher plants, and it is not a product of any evolutionary event, as single-copy regions exist in two equimolar states (Palmer 1983; Walker et al. 2015).

### Phylogenomics analyses

The rapid increase in the amount of complete chloroplast genome sequences during the past decade, provided essential data to elucidate further and resolve phylogenetic relationships among species. Consequently, in a move towards chloroplast phylogenomics, clarification of phylogenetic relationships at higher and lower taxonomic levels have been achieved (Lu et al. 2015; Ma et al. 2014; Wu et al. 2013). The tribe Senecioneae is often subdivided into three subtribes; Senecioninae, Tussilagininae, and Blennospermatinae (Chen et al. 2011). In this study, the multi-gene analysis resulted in a phylogenetic tree whose branches were strongly supported. The five genera of Senecioneae formed two distinct clades that corresponded to two of the three subtribes of Senecioneae including Senecioninae (*Dendrosenecio, Senecio, Jacobaea* and *Pericallis*) and Tussilagininae (*Ligularia*; Fig. 7, Additional file 5: Figure S2). The 11 newly generated species were well placed within the Senecioneae tribe by both ML and BI phylogenetic methods under partitioned and unpartitioned data schemes. The afro-alpine species of *Senecio* are classified in at least five clades of *Senecio* (Kandziora et al. 2016). The present phylogenetic study recovered a monophyletic group with two sub-clades which split *S. keniophytum* and *S. roseiflorus* from *S. purtschelleri, S. moorei* and *S. schweinfurthii.* The relationships among the species of *Senecio* was identical in all data schemes differing only in support of the *S. moorei* and *S. schweinfurthii* relationship (94% and 0.3 PP; Fig. 7). The genus *Jacobaea*, just like *Dendrosenecio*, was previously classified in *Senecio* under section *Jacobaea* (Pelser et al. 2002) but later segregated from *Senecio* based on new insights from molecular phylogeny (Pelser et al. 2006). A sister relationship between *Senecio* and *Jacobaea* was highlighted in this study.

Previously, genetic relationship within *Dendrosenecio* species was shown to be strongly correlated to geographic distance (Knox and Palmer 1995a) as geographically close species were genetically more related than distantly located species. In this study two clades were formed within a monophyletic group of *Dendrosenecio*, one contained *D. johnstonii* and *D. meruensis,* both from Tanzanian mountains. The other clade had species from Kenyan mountains; *D. keniodendron,* and *D. battiscombei* (Mt. Kenya), *D. elgonensis* subsp. *elgonensis* (Mt. Elgon) and *D. brassiciformis*, which was sampled from the Aberdare ranges (Fig. 7a). However, the sister relationships among the Kenyan species was conflicting between the ML and the BI phylogenetic reconstructions. In both ML trees a clear distinction is established concerning geographical (different mountains) and altitudinal (same mountain) variations (Fig. 7a; Additional file 5: Figure S2a), though this correlation is missing in the BI tree (Fig. 7b; Additional file 5: Figure S2b). Therefore, there is need to carry out further analyses, including more species of *Dendrosenecio* from all habitats in order to make a comprehensive conclusion. A majority of the intergeneric relationships defined here were significantly supported and congruent to most of the previous studies based on a few DNA fragments, including the position of *Parastrephia quadrangularis* (Meyen) Cabrera within the species of *Diplostephium* Kunth (Vargas et al. 2017). Therefore, this study strongly underscored the potential of chloroplast genome-scale data in outlining both inter- and intra-generic phylogenetic relationships within the tribe Senecioneae species and in the family at large. Nonetheless, interspecific relationships were weakly supported and therefore, further comprehensive studies that include more taxon sampling are necessary to enhance our understanding of the evolutionary histories of both *Senecio* and *Dendrosenecio.*

## Conclusion

*Dendrosenecio* is a segregate genus to *Senecio.* Despite exhibiting some striking morphologic similarities, a few differences existed based on which the two genera were separated. Initially, controversies arose over the segregation although a consensus was later arrived at. Amplified fragment length polymorphism data distinctly separated the two genera, affirming the earlier decisions. However, lack of or limited molecular resources have impeded further studies on the respective genera. This study generated the first complete chloroplast genome sequences in each genus. Chloroplast genomes in both genera are highly similar in structure, gene composition and synteny, but they significantly differ in size. A chloroplast genome multi-gene dataset revealed three strongly supported clades within the tribe Senecioneae, markedly splitting *Dendrosenecio* from *Senecio*. Ten primers, targeting the ten highly divergent regions in the chloroplast genomes of Senecioneae species, were designed. Also, 25 polymorphic cpSSR in *Senecio* and five in *Dendrosenecio* were identified. The ten divergent hotspots could offer the much-needed DNA barcodes for species identification and phylogenetic reconstructions within the tribe, while the cpSSRs provides potential markers for future population-level research in each respective genus.

## Additional files

**Additional file 1: Table S1.** Details of the Asteraceae species used in the phylogenetic analyses.

**Additional file 2: Table S2.** Details of Relative Synonymous Codon Usage in chloroplast genomes of 11 species of Senecioneae.

**Additional file 3: Table S3.** Number and type of microsatellite repeat motifs in each of the 11 complete chloroplast genomes.

**Additional file 4: Figure S1.** Comparison of sequence arrangement in the chloroplast genomes of five species of Senecioneae (Asteraceae), against *Nicotiana tabacum* as an external reference genome. Conserved orthologs are indicated by locally collinear blocks. Similar blocks among the genomes are coded in one colour and joined by a line. The genes above the line are transcribed in a clockwise direction, those below the line are transcribed towards the counter-clockwise direction.

**Additional file 5: Figure S2.** Phylogenetic relationships of 75 species of Asteraceae inferred from a partitioned chloroplast genome multi-gene dataset using (a) Maximum Likelihood (ML) and (b) Bayesian Inference (BI) methods.

## Data Availability

The datasets generated during the current study are available in the GenBank repository under the accession numbers KY434193–KY434195, MG560049–MG560051 and MH483946–MH483950. All the datasets used for phylogenetic and comparative analyses were downloaded from GenBank, and the accession numbers are provided in the additional files.

## Abbreviations

ITS: internal transcribed spacer
IR: inverted repeat
SSC: small single copy
LSC: large single copy
SSR: simple sequence repeat
CTAB: cetyltrimethylammonium bromide
PCGs: protein-coding genes
RNA: ribonucleic acid
RSCU: relative synonymous codon usage
CUB: codon usage bias
ENc: effective number of codons
ML: maximum likelihood
BS: bootstrap support
BI: Bayesian inference
PP: posterior probability
cpSSRs: chloroplast microsatellites

## References

[CR1] Amiryousefi A, Hyvönen J, Poczai P (2018). IRscope: an online program to visualize the junction sites of chloroplast genomes. Bioinformatics.

[CR2] Beentje HJ, Jeffrey C, Hind DJN, Beentje HJ, Ghazanfar SA (2005). Compositae (Part 3). Flora of tropical east Africa.

[CR3] Bock R, Timmis JN (2008). Reconstructing evolution: gene transfer from plastids to the nucleus. BioEssays.

[CR4] Camacho C, Coulouris G, Avagyan V, Ma N, Papadopoulos J, Bealer K, Madden TL (2009). BLAST+: architecture and applications. BMC Bioinform.

[CR5] Chen YL, Wu ZY, Raven PH, Hong DY (2011). Senecioneae. Flora of China, vol 20–21.

[CR6] Chen S, Zhou Y, Chen Y, Gu J (2018). fastp: an ultra-fast all-in-one FASTQ preprocessor. Bioinformatics.

[CR7] Cheon K-S, Kim K-A, Yoo K-O (2017). The complete chloroplast genome sequences of three *Adenophora* species and comparative analysis with *Campanuloid* species (Campanulaceae). PLoS ONE.

[CR8] Darling AC, Mau B, Blattner FR, Perna NT (2004). Mauve: multiple alignment of conserved genomic sequence with rearrangements. Genome Res.

[CR9] Dempewolf H (2010). Establishing genomic tools and resources for *Guizotia abyssinica* (L.f.) Cass.—the development of a library of expressed sequence tags, microsatellite loci, and the sequencing of its chloroplast genome. Mol Ecol Resour..

[CR10] Dierckxsens N, Mardulyn P, Smits G (2017). NOVOPlasty: de novo assembly of organelle genomes from whole genome data. Nucleic Acids Res.

[CR11] Doorduin L, Gravendeel B, Lammers Y, Ariyurek Y, Chin-A-Woeng T, Vrieling K (2011). The complete chloroplast genome of 17 individuals of pest species *Jacobaea vulgaris*: SNPs, microsatellites and barcoding markers for population and phylogenetic studies. DNA Res.

[CR12] Downie SR, Jansen RK (2015). A comparative analysis of whole plastid genomes from the Apiales: expansion and contraction of the inverted repeat, mitochondrial to plastid transfer of DNA, and identification of highly divergent noncoding regions. Syst Bot.

[CR13] Doyle JJ (1987). A rapid DNA isolation procedure for small quantities of fresh leaf tissue. Phytochem Bull Bot Soc Am.

[CR14] Eckardt NA (2006). Genomic Hopscotch: gene transfer from plastid to nucleus. Plant Cell.

[CR15] Eckardt NA (2007). Chloroplast intron splicing mechanisms. Plant Cell.

[CR16] Edgar RC (2004). MUSCLE: multiple sequence alignment with high accuracy and high throughput. Nucleic Acids Res.

[CR17] Goulding SE, Olmstead RG, Morden CW, Wolfe KH (1996). Ebb and flow of the chloroplast inverted repeat. Mol Gen Genet.

[CR18] Guisinger MM, Kuehl JV, Boore JL, Jansen RK (2011). Extreme reconfiguration of plastid genomes in the Angiosperm family *Geraniaceae*: rearrangements, repeats, and codon usage. Mol Biol Evol.

[CR19] Guo S (2018). Complete chloroplast genome sequence and phylogenetic analysis of *Paeonia ostii*. Molecules.

[CR20] Hedberg O (1957). Afroalpine vascular plants: a taxonomic revision. Symb Bot Upsal.

[CR21] Hegarty MJ, Abbott RJ, Hiscock SJ, Soltis PS, Soltis DE (2012). Allopolyploid speciation in action: the origins and evolution of *Senecio cambrensis*. Polyploidy and genome evolution.

[CR22] Hildebrand M, Hallick RB, Passavant CW, Bourque DP (1988). Trans-splicing in chloroplasts: the *rps* 12 loci of N*icotiana tabacum*. Proc Natl Acad Sci USA.

[CR23] Jansen RK, Ruhlman TA, Bock R, Knoop V (2012). Plastid genomes of seed plants. Genomics of chloroplasts and mitochondria.

[CR24] Jeffrey C, Chen Y-l (1984). Taxonomic studies on the tribe Senecioneae (Compositae) of Eastern Asia. Kew Bull.

[CR25] Jeffrey C, Halliday P, Wilmot-Dear M, Jones SW (1977). Generic and sectional limits in *Senecio* (Compositae): I. Progress report. Kew Bull.

[CR26] Kalyaanamoorthy S, Minh BQ, Wong TKF, von Haeseler A, Jermiin LS (2017). ModelFinder: fast model selection for accurate phylogenetic estimates. Nat Methods.

[CR27] Kandziora M, Kadereit JW, Gehrke B (2016). Frequent colonization and little in situ speciation in *Senecio* in the tropical alpine-like islands of eastern Africa. Am J Bot.

[CR28] Kim K-J, Choi K-S, Jansen RK (2005). Two chloroplast DNA inversions originated simultaneously during the early evolution of the sunflower family (Asteraceae). Mol Biol Evol.

[CR29] Knox EB, Beentje HJ, Jeffrey C, Hind DJN (2005). *Dendrosenecio*. Flora of Tropical East Africa, Compositae (part 3).

[CR30] Knox EB (2014). The dynamic history of plastid genomes in the *Campanulaceae* sensu lato; is unique among angiosperms. Proc Natl Acad Sci USA.

[CR31] Knox EB, Palmer JD (1995). Chloroplast DNA variation and the recent radiation of the giant senecios (Asteraceae) on the tall mountains of eastern Africa. Proc Natl Acad Sci USA.

[CR32] Knox EB, Palmer JD (1995). The origin of *Dendrosenecio* within the Senecioneae (Asteraceae) based on chloroplast DNA evidence. Am J Bot.

[CR33] Kumar S, Hahn FM, McMahan CM, Cornish K, Whalen MC (2009). Comparative analysis of the complete sequence of the plastid genome of *Parthenium argentatum* and identification of DNA barcodes to differentiate *Parthenium* species and lines. BMC Plant Biol.

[CR34] Lanfear R, Frandsen PB, Wright AM, Senfeld T, Calcott B (2017). PartitionFinder 2: new methods for selecting partitioned models of evolution for molecular and morphological phylogenetic analyses. Mol Biol Evol.

[CR35] Lee J, Lee H, Lee S-C, Sung SH, Kang JH, Lee TJ, Yang T-J (2016). The complete chloroplast genome sequence of *Ligularia fischeri* (Ledeb.) Turcz. (Asteraceae). Mitochondrial DNA Part B.

[CR36] Letunic I, Bork P (2016). Interactive tree of life (iTOL) v3: an online tool for the display and annotation of phylogenetic and other trees. Nucleic Acids Res.

[CR37] Lohse M, Drechsel O, Bock R (2007). OrganellarGenomeDRAW (OGDRAW): a tool for the easy generation of high-quality custom graphical maps of plastid and mitochondrial genomes. Curr Genet.

[CR38] Lu J-M, Zhang N, Du X-Y, Wen J, Li D-Z (2015). Chloroplast phylogenomics resolves key relationships in ferns. J Syst Evol..

[CR39] Lu C, Shen Q, Yang J, Wang B, Song C (2016). The complete chloroplast genome sequence of Safflower (*Carthamus tinctorius* L.). Mitochondrial DNA A DNA Mapp Seq Anal..

[CR40] Ma PF, Zhang YX, Zeng CX, Guo ZH, Li DZ (2014). Chloroplast phylogenomic analyses resolve deep-level relationships of an intractable bamboo tribe Arundinarieae (Poaceae). Syst Biol.

[CR41] Mabberley DJ (1973). Evolution in the giant groundsels. Kew Bull.

[CR42] Marechal A, Brisson N (2010). Recombination and the maintenance of plant organelle genome stability. New Phytol.

[CR43] Martin W, Stoebe B, Goremykin V, Hansmann S, Hasegawa M, Kowallik KV (1998). Gene transfer to the nucleus and the evolution of chloroplasts. Nature.

[CR44] Milton JJ (2009). Phylogenetic analyses and taxonomic studies of Senecioninae: southern African *Senecio* section *Senecio*.

[CR45] Morton BR (1993). Chloroplast DNA codon use: evidence for selection at the *psb* A locus based on tRNA availability. J Mol Evol.

[CR46] Nguyen L-T, Schmidt HA, von Haeseler A, Minh BQ (2015). IQ-TREE: a fast and effective stochastic algorithm for estimating maximum-likelihood phylogenies. Mol Biol Evol.

[CR47] Nie X (2012). Complete chloroplast genome sequence of a major invasive species, Crofton weed (*Ageratina adenophora*). PLoS ONE.

[CR48] Nie X, Deng P, Feng K, Liu P, Du X, You FM, Weining S (2014). Comparative analysis of codon usage patterns in chloroplast genomes of the Asteraceae family. Plant Mol Biol Rep.

[CR49] Niu Z, Xue Q, Wang H, Xie X, Zhu S, Liu W, Ding X (2017). Mutational biases and GC-biased gene conversion affect GC content in the plastomes of *Dendrobium* genus. Int J Mol Sci.

[CR50] Nordenstam B (1978). Taxonomic studies in the tribe Senecioneae (Compositae). Opera Bot Lund.

[CR51] Nordenstam B, Kadereit JW, Jeffrey C, Kubitzki K (2007). Tribe Senecioneae. The families and genera of vascular plants.

[CR52] Nordenstam B, Pelser PB, Kadereit JW, Watson LE, Funk VA, Susanna A, Stuessy TF, Bayer RJ (2009). Senecioneae. Systematics, evolution, and biogeography of composiate.

[CR53] Palmer JD (1983). Chloroplast DNA exists in two orientations. Nature.

[CR54] Pelser PB, Gravendeel B, van der Meijden R (2002). Tackling speciose genera: species composition and phylogenetic position of *Senecio* sect. *Jacobaea* (Asteraceae) based on plastid and nrDNA sequences. Am J Bot.

[CR55] Pelser PB, Veldkamp JF, van der Meijden R (2006). New combinations in *Jacobaea* Mill. (Asteraceae–Senecioneae). Compos Newsl.

[CR56] Pelser PB, Nordenstam B, Kadereit JW, Watson LE (2007). An ITS phylogeny of tribe Senecioneae (Asteraceae) and a new delimitation of *Senecio* L. Taxon.

[CR57] Plant AL, Gray JC (1988). Introns in chloroplast protein-coding genes of land plants photosynthesis. Research.

[CR58] Pouchon C, Fernández A, Nassar JM, Boyer F, Aubert S, Lavergne S, Mavárez J (2018). Phylogenomic analysis of the explosive adaptive radiation of the *Espeletia* complex (Asteraceae) in the tropical Andes. Syst Biol.

[CR59] Ravi V, Khurana JP, Tyagi AK, Khurana P (2008). An update on chloroplast genomes. Plant Syst Evol.

[CR60] Ronquist F (2012). MrBayes 3.2: efficient Bayesian phylogenetic inference and model choice across a large model space. Syst Biol.

[CR61] Rozas J, Ferrer-Mata A, Sanchez-DelBarrio JC, Guirao-Rico S, Librado P, Ramos-Onsins SE, Sanchez-Gracia A (2017). DnaSP 6: DNA sequence polymorphism analysis of large data sets. Mol Biol Evol.

[CR62] Salih M, Majeský Ľ, Schwarzacher T, Gornall R, Heslop-Harrison P (2017). Complete chloroplast genomes from apomictic *Taraxacum* (Asteraceae): identity and variation between three microspecies. PLoS ONE.

[CR63] Sharp PM, Li W-H (1987). The codon adaptation index-a measure of directional synonymous codon usage bias, and its potential applications. Nucleic Acids Res.

[CR64] Shaw J (2005). The tortoise and the hare II: relative utility of 21 noncoding chloroplast DNA sequences for phylogenetic analysis. Am J Bot.

[CR65] Shen X (2017). Complete chloroplast genome sequence and phylogenetic analysis of the medicinal plant *Artemisia annua*. Molecules.

[CR66] Shinozaki K (1986). The complete nucleotide sequence of the tobacco chloroplast genome: its gene organization and expression. EMBO J.

[CR67] Tautz D, Renz M (1984). Simple sequences are ubiquitous repetitive components of eukaryotic genomes. Nucleic Acids Res.

[CR68] Thiel T, Michalek W, Varshney R, Graner A (2003). Exploiting EST databases for the development and characterization of gene-derived SSR-markers in barley (*Hordeum vulgare* L.). Theor Appl Genet.

[CR69] Tillich M, Lehwark P, Pellizzer T, Ulbricht-Jones ES, Fischer A, Bock R, Greiner S (2017). GeSeq—versatile and accurate annotation of organelle genomes. Nucleic Acids Res.

[CR70] Timmis JN, Ayliffe MA, Huang CY, Martin W (2004). Endosymbiotic gene transfer: organelle genomes forge eukaryotic chromosomes. Nat Rev Genet.

[CR71] Tonti-Filippini J, Nevill PG, Dixon K, Small I (2017). What can we do with 1000 plastid genomes?. Plant J..

[CR72] Uddin A (2017). Indices of codon usage bias. J Proteom Bioinform.

[CR73] Untergasser A, Cutcutache I, Koressaar T, Ye J, Faircloth BC, Remm M, Rozen SG (2012). Primer3-new capabilities and interfaces. Nucleic Acids Res.

[CR74] Vargas OM, Ortiz EM, Simpson BB (2017). Conflicting phylogenomic signals reveal a pattern of reticulate evolution in a recent high-Andean diversification (Asteraceae: Astereae: *Diplostephium*). New Phytol.

[CR75] Walker JF, Zanis MJ, Emery NC (2014). Comparative analysis of complete chloroplast genome sequence and inversion variation in *Lasthenia burkei* (Madieae, Asteraceae). Am J Bot.

[CR76] Walker JF, Jansen RK, Zanis MJ, Emery NC (2015). Sources of inversion variation in the small single copy (SSC) region of chloroplast genomes. Am J Bot.

[CR77] Wang M (2015). Comparative analysis of Asteraceae chloroplast genomes: structural organization, RNA editing and evolution. Plant Mol Biol Rep.

[CR78] Wang B, Li M, Yuan Y (2019). The complete chloroplast genome of *Pericallis hybrida* (Asteridae). Mitochondrial DNA Part B.

[CR79] Wicke S, Schneeweiss GM, dePamphilis CW, Müller KF, Quandt D (2011). The evolution of the plastid chromosome in land plants: gene content, gene order, gene function. Plant Mol Biol.

[CR80] Wright F (1990). The ‘effective number of codons’ used in a gene. Gene.

[CR81] Wu C-S, Chaw S-M, Huang Y-Y (2013). Chloroplast phylogenomics indicates that *Ginkgo biloba* is sister to Cycads. Genome Biol Evol.

[CR82] Wu M-l, Li Q, Xu J, Li X-w (2018). Complete chloroplast genome of the medicinal plant *Amomum compactum*: gene organization, comparative analysis, and phylogenetic relationships within Zingiberales. Chin Med.

[CR83] Yurina NP, Sharapova LS, Odintsova MS (2017). Structure of plastid genomes of photosynthetic eukaryotes. Biochemistry.

[CR84] Zhang D, Gao F, Li WX, Jakovlić I, Zou H, Zhang J, Wang GT (2018). PhyloSuite: an integrated and scalable desktop platform for streamlined molecular sequence data management and evolutionary phylogenetics studies. bioRxiv.

