# Supplementary material for: Comparative genomics of 11 complete chloroplast genomes of Senecioneae (Asteraceae) species: DNA barcodes and phylogenetics
Source: Bot Stud. 2019 Aug 22;60:17. doi: 10.1186/s40529-019-0265-y (PMC6706487; doi:10.1186/s40529-019-0265-y)
Supplement: Supplementary file 3 — Additional file 3: Table S3. Number and type of microsatellite repeat motifs in each of the 11 complete chloroplast genomes. [file 40529_2019_265_MOESM3_ESM.docx]

| **Repeat type** | **S. keniophytum** | **S. moorei** | **S. purtschelleri** | **S. schweinfurthii** | **S. roseiflorus** | **D. johnstonii** | **D. keniodendron** | **D. meruensis** | **D. battiscombei** | **D. brassiciformis** | **D. keniensis** |
| --- | --- | --- | --- | --- | --- | --- | --- | --- | --- | --- | --- |
| A/T | 35 | 34 | 33 | 32 | 40 | 33 | 26 | 36 | 29 | 34 | 29 |
| C/G | 0 | 0 | 0 | 0 | 0 | 1 | 0 | 0 | 0 | 0 | 0 |
| AC/GT | 0 | 1 | 1 | 1 | 1 | 0 | 0 | 0 | 0 | 0 | 0 |
| AT/AT | 8 | 8 | 8 | 8 | 8 | 11 | 10 | 11 | 10 | 10 | 10 |
| AAG/CTT | 2 | 2 | 2 | 2 | 2 | 1 | 1 | 1 | 1 | 1 | 1 |
| AAT/ATT | 7 | 8 | 8 | 9 | 8 | 4 | 4 | 4 | 4 | 4 | 4 |
| ACT/AGT | 1 | 0 | 0 | 0 | 1 | 0 | 0 | 0 | 0 | 0 | 0 |
| AAAG/CTTT | 1 | 1 | 1 | 1 | 1 | 1 | 1 | 1 | 1 | 1 | 1 |
| AAAT/ATTT | 6 | 6 | 6 | 6 | 6 | 4 | 4 | 4 | 4 | 4 | 4 |
| AACT/AGTT | 0 | 0 | 0 | 0 | 0 | 1 | 1 | 1 | 1 | 1 | 1 |
| AATG/ATTC | 0 | 0 | 0 | 0 | 0 | 1 | 1 | 1 | 1 | 1 | 1 |
| AGAT/ATCT | 0 | 0 | 0 | 0 | 0 | 1 | 1 | 1 | 1 | 1 | 1 |
| AATC/ATTG | 1 | 1 | 1 | 1 | 1 | 0 | 0 | 0 | 0 | 0 | 0 |
| AATT/AATT | 3 | 1 | 1 | 1 | 2 | 0 | 0 | 0 | 0 | 0 | 0 |
| AAAAT/ATTTT | 0 | 1 | 1 | 1 | 0 | 2 | 2 | 3 | 2 | 2 | 0 |
| AATCT/AGATT | 1 | 0 | 0 | 0 | 0 | 0 | 0 | 0 | 0 | 0 | 0 |
| AATTC/AATTG | 1 | 0 | 0 | 0 | 0 | 0 | 0 | 0 | 0 | 0 | 0 |
| AAGTAC/ACTTGT | 2 | 2 | 2 | 2 | 0 | 0 | 0 | 0 | 0 | 0 | 0 |
| AGCTAT/AGCTAT | 0 | 0 | 0 | 0 | 0 | 1 | 0 | 0 | 0 | 0 | 0 |

**Table S3** Number and type of microsatellite repeat motifs in each of the 11 complete chloroplast genomes.

**Total 68 65 64 64 70 61 51 63 54 59**
