# Supplementary figures and images for: Comparative genomics of 11 complete chloroplast genomes of Senecioneae (Asteraceae) species: DNA barcodes and phylogenetics
Source: Bot Stud. 2019 Aug 22;60:17. doi: 10.1186/s40529-019-0265-y (PMC6706487; doi:10.1186/s40529-019-0265-y)

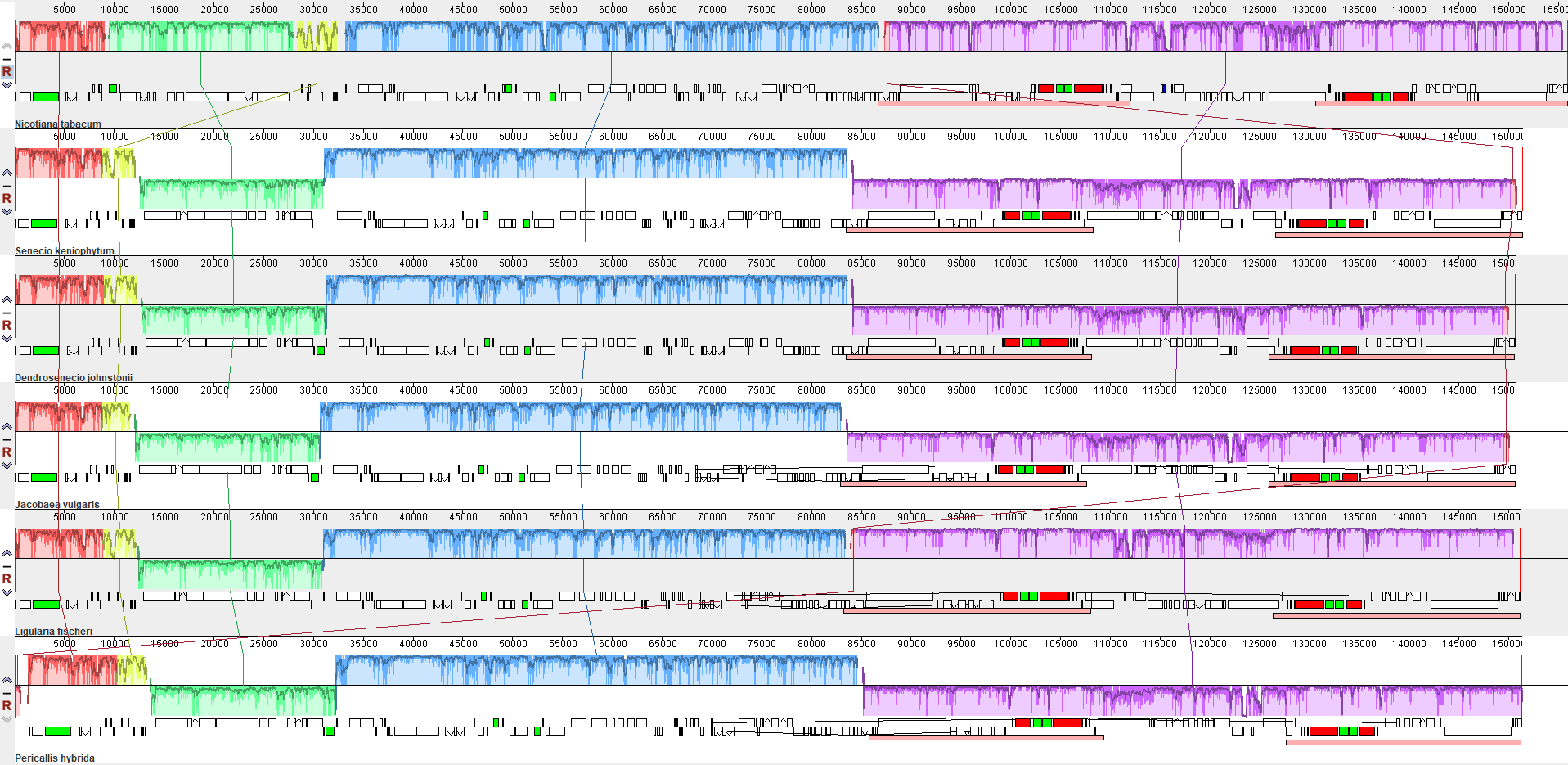

Supplement: Supplementary file 4 — Additional file 4: Figure S1. Comparison of sequence arrangement in the chloroplast genomes of five species of Senecioneae (Asteraceae), against Nicotiana tabacum as an external reference genome. Conserved orthologs are indicated by locally collinear blocks. Similar blocks among the genomes are coded in one colour and joined by a line. The genes above the line are transcribed in a clockwise direction, those below the line are transcribed towards the counter-clockwise direction. [file 40529_2019_265_MOESM4_ESM.png]

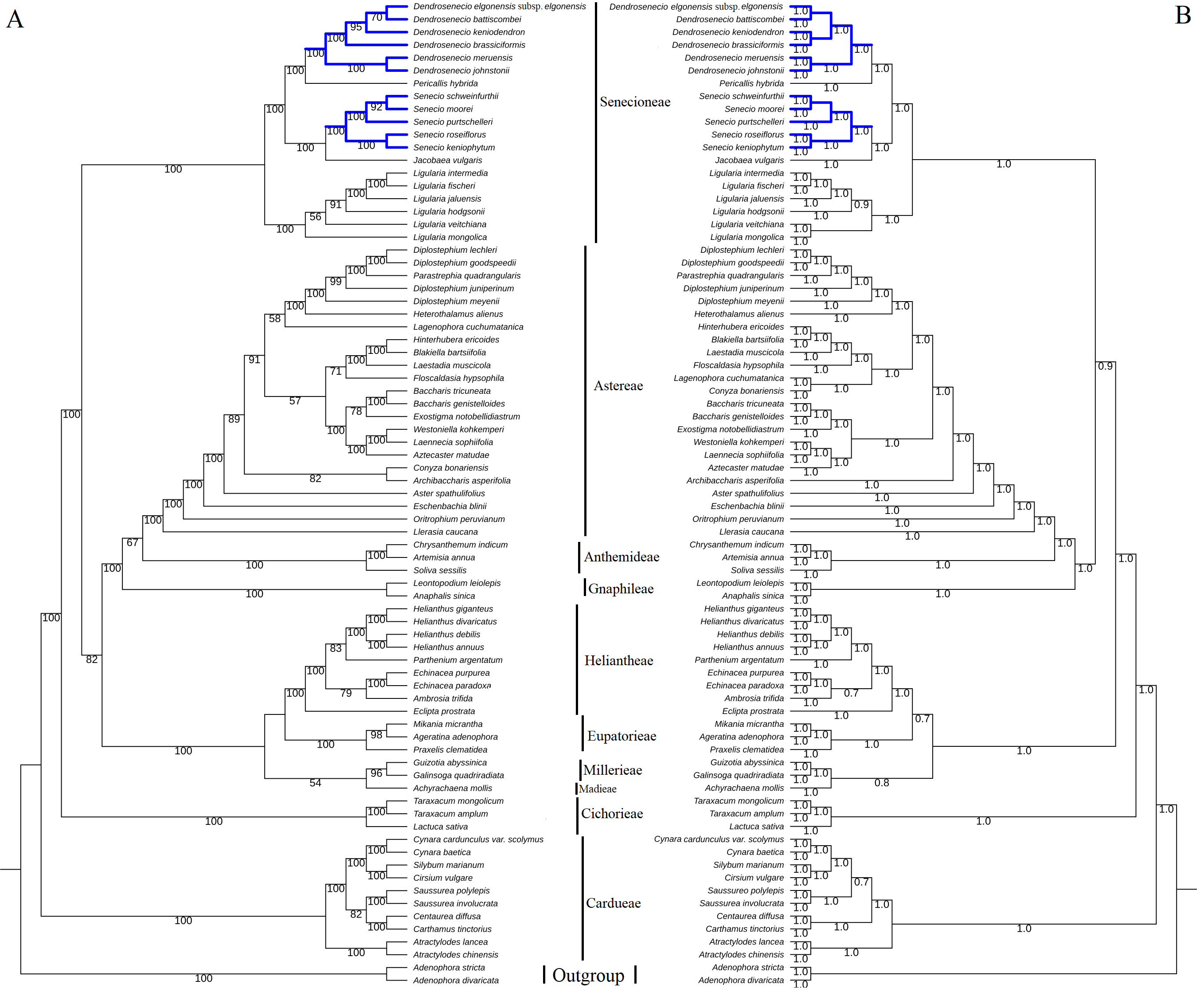

Supplement: Supplementary file 5 — Additional file 5: Figure S2. Phylogenetic relationships of 75 species of Asteraceae inferred from a partitioned chloroplast genome multi-gene dataset using (a) Maximum Likelihood (ML) and (b) Bayesian Inference (BI) methods. [file 40529_2019_265_MOESM5_ESM.png]
